# Physioxia Stimulates Extracellular Matrix Deposition and Increases Mechanical Properties of Human Chondrocyte-Derived Tissue-Engineered Cartilage

**DOI:** 10.3389/fbioe.2020.590743

**Published:** 2020-11-13

**Authors:** James E. Dennis, George Adam Whitney, Jyoti Rai, Russell J. Fernandes, Thomas J. Kean

**Affiliations:** ^1^Benaroya Research Institute, Seattle, WA, United States; ^2^Department of Orthopaedics and Sports Medicine, University of Washington, Seattle, WA, United States

**Keywords:** tissue-engineered cartilage, type II collagen, biomechanical testing, articular cartilage, chondrocyte, chondrogenesis, collagen cross linking, hypoxia

## Abstract

Cartilage tissue has been recalcitrant to tissue engineering approaches. In this study, human chondrocytes were formed into self-assembled cartilage sheets, cultured in physiologic (5%) and atmospheric (20%) oxygen conditions and underwent biochemical, histological and biomechanical analysis at 1- and 2-months. The results indicated that sheets formed at physiological oxygen tension were thicker, contained greater amounts of glycosaminoglycans (GAGs) and type II collagen, and had greater compressive and tensile properties than those cultured in atmospheric oxygen. In all cases, cartilage sheets stained throughout for extracellular matrix components. Type II-IX-XI collagen heteropolymer formed in the neo-cartilage and fibrils were stabilized by trivalent pyridinoline cross-links. Collagen cross-links were not significantly affected by oxygen tension but increased with time in culture. Physiological oxygen tension and longer culture periods both served to increase extracellular matrix components. The foremost correlation was found between compressive stiffness and the GAG to collagen ratio.

## Introduction

Cartilage tissue has very poor intrinsic repair capacity. While osteoarthritis is a complex, multifaceted disease, cartilage degradation is a core component. Autologs chondrocyte implantation and matrix assisted autologs chondrocyte implantation have provided relief to patients but commonly result in fibrocartilage repair (Shekkeris et al., 2012). Tissue engineering could potentially address this through *in vitro* culture methods to produce functional hyaline cartilage tissue, with several examples currently in clinical trials (Kwon et al., 2019). We, and others, have investigated media supplements and growth factors to improve the expansion and re-differentiation of the expanded chondrocytes (Vunjak-Novakovic et al., 1999; Enochson et al., 2012; Makris et al., 2013a; Dennis et al., 2020).

Cartilage, being avascular, is normally exposed to low levels of oxygen (2–5%), meaning that this would be physiological conditions (Pattappa et al., 2019). It is increasingly apparent that physiological oxygen tension should be the standard culture method to grow tissue engineered human articular cartilage whether it be from mesenchymal stem cells (Pattappa et al., 2019), articular chondrocytes (Kean and Dennis, 2012a,b, 2013, 2015; Kean et al., 2013, 2015, 2016, 2019; Markway et al., 2013; Bianchi et al., 2017) or chondroprogenitors (Anderson et al., 2018). The selection of cell type for engineered tissue raises some interesting issues, mesenchymal stromal cells (MSC) commonly progress to hypertrophy (Mueller and Tuan, 2008) as do iPSCs driven down the mesenchymal pathway (Adkar et al., 2019), a negative scenario for the production of hyaline cartilage. Hypertrophy has been reduced but not eliminated during MSC culture (Gawlitta et al., 2012) and subcutaneous implants by pharmacological and/or culture-dependent methods (Diederichs et al., 2019). This study focuses on the use of human articular chondrocytes derived from discarded total joint replacement tissue as both a clinically relevant and non-hypertrophic cell source (in our hands). We, and others, have focused on scaffold-free self-assembly of tissue-engineered cartilage, as significant similarities to native tissue structure can be achieved (Weidenbecher et al., 2008; Gilpin et al., 2010; Whitney et al., 2012; Kean et al., 2016; Anderson et al., 2018; Dennis et al., 2018; Huang et al., 2018; Mansour et al., 2018; Whitney et al., 2018). Significant expansion, up to eight population doublings, of human chondrocytes while maintaining their differentiation capacity has been achieved through their culture on devitalized synoviocyte matrix (Kean and Dennis, 2015). Using these methods, we produced sheets of human articular chondrocyte-derived cartilage and investigated the effect of low, physiological, oxygen tension and duration of culture on cartilage quality in terms of biomechanics and biochemical content. It was hypothesized that physiological oxygen tension and increased culture duration would improve the mechanical properties of the tissue engineered cartilage through an increase in extracellular matrix content. This study adds to the current body of literature by expanding the donor pool, focuses on chondrocytes isolated from total joint replacement tissue as a cell source, and includes analysis of cartilage-typic collagen heteropolymer formation, collagen cross-links and tensile properties of tissue engineered cartilage.

## Materials and Methods

### Cell and Tissue Culture

**FIGURE 1:**
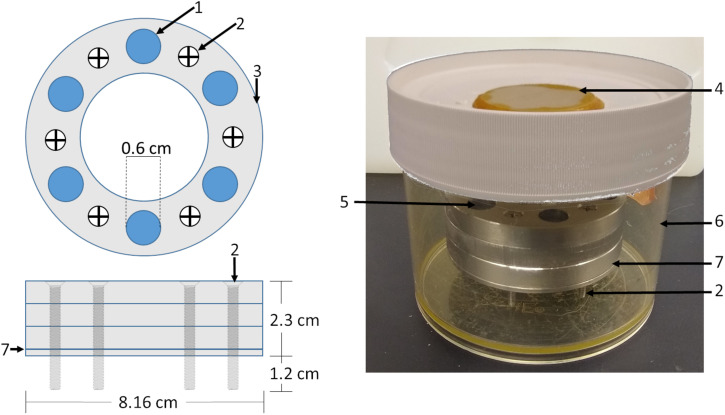
Biochamber model and setup. A circular biochamber design with a 1.13 cm^2^ cell seeding area (1) was used with three seeding chambers (3) stacked on top of each other giving a 2.1 ml seeding volume. Stainless steel screws (2) were used to raise the biochambers 1.2 cm allowing media access to the cell sheet through the polyester membrane (7) sandwiched between the bottom 0.2 cm plate and the seeding chambers. Biochambers were assembled and placed in Nalgene containers (6) with a ceramic filter on the lid (4). Defined chondrogenic media was added to the level of the membrane, cells seeded, then media added to 0.2 cm below the top of the topmost seeding chamber.

Human articular chondrocytes were thawed from frozen stocks obtained from discarded surgical tissue of patients (*n* = 6) undergoing total joint replacement collected with IRB approval. Human articular chondrocytes were expanded under physiological oxygen tension (5%; Physioxia) on synoviocyte derived extracellular matrix in growth media (DMEM-LG supplemented with 10% FBS and 1% penicillin/streptomycin) (Kean and Dennis, 2015). Synoviocyte derived extracellular matrix was generated using the method described in Kean et al. (2015) Briefly, synoviocytes from porcine knees were isolated using sequential digest with hyaluronidase, trypsin/EDTA then collagenase. Synoviocytes were expanded for two passages then seeded at 6,000 cells/cm^2^, grown until 70–80% confluence (~5 days) in DMEM-LG + 10% FBS + 1% pen/strep, then switched to ascorbate containing media (DMEM-LG + 10% FBS + 1% pen/strep + 50 μM ascorbate-2-phosphate for a further 7 days. At this point, cells were washed with PBS then the flask flash frozen on dry ice-cooled ethanol. Dry ice-cooled ethanol was then pipetted into the flask to devitalize the synoviocytes, removed and evaporated. Devitalized synoviocyte matrix covered flasks were stored at 4°C until use (<6-months). At the end of first passage (six experiments) or second passage (two experiments), cells were trypsinized and seeded at high density (4.4 × 10^6^ cells/cm^2^) in a custom stainless steel biochamber (Figure 1) (Whitney et al., 2012). Partner biochambers for large (16 cm^2^) (Whitney et al., 2012), seeded at the same cell density, were made in several experiments for other studies. All assays were performed on the 1.13 cm^2^ pieces of tissue engineered cartilage (Figure 1). Biochambers were assembled and sterilized by autoclave. The polyester membrane was coated with fibronectin (8 μg/cm^2^ in PBS) and allowed to dry in a biosafety cabinet. Biochambers were used much as previously described but with a stacked, 1.1 cm^2^ circular seeding chamber with longer screws creating greater space above and below the chamber for media exchange (Figure 1). Biochambers were cultured at either atmospheric oxygen tension (20%; Atm O_2_) or Physioxia (5% O_2_) in defined chondrogenic medium [DMEM-HG containing 1% ITS + premix, dexamethasone (100 nM), ascorbate-2-phosphate (120 μM), 1% MEM NEAA, 1% Pen/Strep, 1% glutamax, TGFβ1 (10 ng/ml)] for 21 days (3-weeks) and 46–56 days (7-weeks) with 50% media changes every other day. All cultures were grown at 37°C with 5% CO_2_ in a humidified atmosphere. After 5-days in static culture, all biochambers were put on a rotating shaker (60 RPM). At the end of culture, three 5mm skin biopsy punches were taken for mechanical assessment (equilibrium modulus and tensile modulus). Remaining tissue was assessed by biochemical [glycosaminoglycan (GAG), DNA, hydroxyproline (HDP), and collagen cross-link content (hydroxylysyl pyridinoline + lysyl pyridinoline (HP + LP); moles/mole collagen)] and histological assays.

### Biochemical Assays

Glycosaminoglycan, DNA and HDP assays were conducted much as previously described (Kean et al., 2016). Briefly, tissue engineered cartilage was digested with papain solution (25 μg/mL papain, 2 mM cysteine, 50 mM sodium phosphate, and 2 mM EDTA adjusted to pH 6.5 [all from Sigma-Aldrich]) at 65°C for 3 h. Digested tissue was then split between the HDP assay and GAG/DNA assays. For the GAG/DNA portion of the assay, papain digested samples were inactivated by sodium hydroxide (2 volumes 0.1 M NaOH) then neutralized with acidified phosphate buffer (2 volumes 100 mM sodium phosphate pH 7.2 acidified with 0.1 M HCl). GAG was assessed using safranin-O: Neutralized samples were incubated with Safranin-O solution (0.05% in 50 mM sodium acetate) on a dot blot apparatus (BioRad) with a 0.45 μm nitrocellulose membrane in duplicate. Dots were punched from the membrane and incubated in cetylpyridinium chloride solution (10%, Alpha Aesar) at 37°C for 20 min. The extract was then transferred in triplicate to a clear 96-well microplate and absorbance measured (536 nm; Tecan M200). GAG concentrations were calculated from a standard curve produced with chondroitin sulfate (Seikagaku Chemicals). DNA was assessed from neutralized samples using buffered Hoechst solution (0.667 μg/ml in 0.2 M pH 8.0 phosphate buffer; 33258; Sigma-Aldrich). Neutralized digest (20μl) was transferred to a black 96-well plate in duplicate and Hoechst solution added (100 μl) then fluorescence read (Ex 365 nm, Em 460 nm; Tecan M200). DNA content was calculated from a standard curve (calf thymus DNA; Sigma-Aldrich) made in neutralized papain buffer. For the assessment of HDP, papain digested samples were acid hydrolyzed overnight (10:1 vol/vol, 6 M HCl, 110°C). Acid hydrolysate was then evaporated to dryness by incubation at 70°C 1–2 days. Samples and HDP standards were then resuspended in ddH_2_O, mixed with 1 volume copper sulfate (0.15 M), 1 volume NaOH (2.5 M) and incubated (50°C, 5 min). Samples were then oxidized by incubation with hydrogen peroxide (1 volume, 6% H_2_O_2_; 50°C, 10 min). To this solution, 4 volumes of sulfuric acid were added (1.5 M H_2_SO_4_) then reacted with Ehrlich’s reagent (2 volumes: 10% w/v 4-dimethylamino benzaldehyde in 60% isopropanol, 26% perchloric acid, 14% MΩ water) at 70°C for 16 min. After cooling, samples and standard absorbance was read on a plate reader (505 nm, Tecan M200). Collagen content was estimated from HDP concentration by a conversion factor of 7.6 (Venn and Maroudas, 1977).

### Collagen Cross-Link Analysis

Samples were dried, weighed and then acid hydrolyzed in 6 M HCl, 110°C for 24 h. HP and LP cross-linking residues were resolved and quantified by C-18 reverse phase HPLC with fluorescence detection (excitation 297 nm, emission 396 nm) and total collagen content was determined as described (Fernandes et al., 1998).

### Collagen Heteropolymer Analysis

Unused portions of the samples used for mechanical analyses were used to qualitatively fingerprint cross-linked collagen types by western blots. The heteropolymeric collagen network formed in the samples was depolymerized in equal volumes of 0.5 M acetic acid containing 100 μg/ml pepsin for 18h at 4°C. Unused portions of neo-cartilage after mechanical tests were used for SDS-PAGE, these were normalized by wet weight within oxygen tension. Equal aliquots of solubilized collagen were analyzed by SDS-PAGE and the separated collagen chains visualized by Coomassie blue staining. Pepsin-extracted type II collagen from human articular cartilage was used as a control. The separated collagen chains were also blotted onto PVDF membranes and probed with mAb 1C10 to identify α1(II) chains and with mAb 10F2, pAb 5890, mAb 2B4 to identify collagen chains cross-linked to the *C*-telopeptide of α1(II), to the *N*-telopeptide of α1(XI) chains and to the α1(IX) chains, respectively (Fernandes et al., 2003). As we have described before, this validates if a heteropolymer of type II and type XI collagen had formed (Murdoch et al., 2016).

### Histological Analysis

Samples were fixed in neutral buffered formalin overnight at 4°C then switched to PBS at 4°C until embedded. Samples were embedded by sequential dehydration in graded ethanols, xylene, and paraffin. Paraffin sections (8 μm) were deparaffinized and hydrated before staining with safranin-O (Sigma-Aldrich) for GAG with a Fast Green (AA16520-06, Alfa Aesar) counterstain. For immunohistochemistry, hydrated sections were subjected to antigen retrieval by pronase (1 mg/ml in PBS containing 5 mM calcium chloride; Sigma-Aldrich) incubation for 10 min at room temperature. Primary antibodies against type I collagen (631703, MP Biomedical, 1:1,000), type II collagen (DSHB II-II6B3 cell culture supernatant, 1:500) and type X collagen (kind gift of Gary Gibson, Henry Ford Hospital, Detroit, MI, United States; 1:500) were incubated with tissue sections at 4°C overnight. Sections were then rinsed and stained with secondary antibody (biotinylated horse anti-mouse; Gibco; BA2000; 1:2,000) for 1 h at room temperature before rinsing and incubation with streptavidin-HRP (SNN1004, Invitrogen, 1:5,000) 30 min at room temperature. Detection was then made with VIP substrate (Vectashield) by incubation at room temperature for 10 min. Slides were rinsed and counterstained with Fast Green before mounting.

### Mechanical Analysis

Samples were thawed in PBS solution equilibrated to room temperature for at least 30 min. Punches were measured three times with digital calipers to assess thickness. Compressive equilibrium moduli were determined as previously described (Lima et al., 2007). Briefly, after an initial tare load of 0.2 N, 4 sequential strains of 5, 10, 15, and 20% were applied, with a stress-equilibration period of 30 min between each strain step. The stress measured at the end of each strain period was taken as the apparent stress at the corresponding strain level. The equilibrium modulus was determined as the slope between the apparent stress and the strain.

**FIGURE 2:**
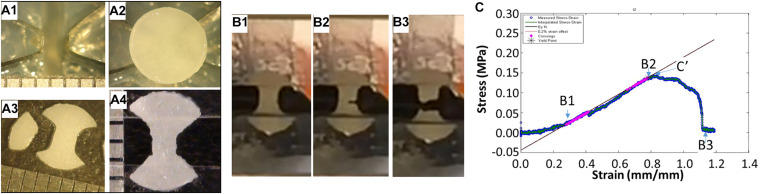
Tissue engineered cartilage sample preparation and tensile testing. Panel **(A)** shows the custom manufactured dog bone punch **(A1)**, the 5 mm punched tissue-engineered human cartilage sheet on the dogbone punch **(A2)**, the resulting dogbone **(A3)**, and the dogbone fixed to the OHP film adapter to insert into the grips of the mechanical testing device. Panel **(B)** shows sequential images of a piece of tissue-engineered human cartilage being tested in tension to failure. Panel **(C)** shows the stress strain curve of the cartilage piece with approximate points shown in the graph of the images in **(B)**, with **(B2)** indicating the yield stress, in this case close to the ultimate yield stress **(C′)**, and the best fit line showing the tensile Young’s modulus.

To test elastic tensile Young’s moduli, a custom dogbone punch was made from skin biopsy punches and punches taken from the 5 mm punch (Figure 2A). Custom holders were made from overhead projector sheets (Supplementary File 1) and dogbones attached using cyanoacrylate glue (Figure 2A4), tissue hydration was maintained with PBS. Tissues were stretched to failure (Figures 2B,C) and the tensile Young’s modulus, ultimate tensile stress and yield stress calculated. Residual pieces of cartilage from the dogbone punch were used for collagen typing and heteropolymer analysis.

### Samples and Statistics

Eight separate experiments were performed with chondrocytes from six human donors. For the analysis of GAG/DNA/HDP, three samples per sheet were taken from up to two replicate sheets and assayed in duplicate. Each experiment was averaged and data shown represents the average for the experiment. When sheets were too flimsy to be manipulated, GAG/DNA/HDP was not performed (three experiments, three donors, five data points, all Atm O_2_). A mixed-effects model was used to analyze log transformed values (GAG/DNA and Collagen/DNA) and collagen crosslink density with Sidak’s multiple comparisons test (GraphPad Prism, V8.4.2).

For mechanical tests two sheets were made for each donor and 2–3 5 mm biopsy punches were taken from each sheet. Punch thickness was measured with digital calipers three times and the average value taken for the thickness. Thickness data was analyzed by a mixed-effects model with Sidak’s multiple comparisons test (GraphPad Prism, V8.4.2). Punches that were incomplete circles, curled too much to get a flat sheet in testing were excluded from analysis. Atmospheric oxygen tension sheets from donors that were insufficiently sturdy or were too thin to be manipulated (2 of 6) have the imputed compressive modulus value of the weakest sheet tested (0.3129 kPa). All data represent the average of the samples tested (*n* = 1–4) for each independent experiment (*n* = 8). Data were analyzed by repeated measures two-way ANOVA with Sidak’s multiple comparisons test (GraphPad Prism, V8.4.2). Four donors were included in the tensile testing experiments, two of which failed to form sheets that were sturdy enough to be tested when cultured at Atm O_2_. Data were analyzed by two-way ANOVA with Sidak’s multiple comparisons test (GraphPad Prism, V8.4.2).

## Results

**FIGURE 3:**
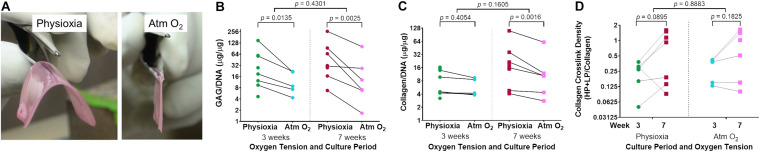
Extracellular matrix deposition in tissue-engineered human cartilage sheets. Sheets grown under Physioxia are more easily manipulated than those grown in Atm O_2_; an example of a large (16 cm^2^) sheet from a single donor grown in Physioxia or Atm O_2_ at 7-weeks **(A)**. Cell normalized glycosaminoglycan (GAG/DNA) content of cartilage from samples grown at Physioxia and Atm O_2_ are shown at 3- and 7-weeks **(B)**. Cell normalized total collagen content of tissue-engineered cartilage when grown in Physioxia and Atm O_2_ at 3 and 7 weeks is shown **(C)**. Trivalent collagen cross-link density (HP + LP/Collagen) at 3- and 7-weeks under both Physioxia and Atm O_2_ are shown **(D)**. Note, all measures are shown on a log (base 2) scale. The average of data for each experiment is shown by a symbol with lines connecting an experiment, six donors, eight experiments.

Handleable tissue-engineered human cartilage sheets formed in all eight experiments in Physioxia vs five of eight experiments at week 3 and six of eight experiments by week 7 at Atm O_2_ (Figure 3).

### Biochemical Assays

There was a significant increase in extracellular matrix content, both in terms of GAG/DNA (Figure 3B) and collagen/DNA (Figure 3C) in human tissue-engineered cartilage sheets when cultured in Physioxia vs Atm O_2_. This increase in GAG/DNA was not significantly affected by time in culture (Figure 3B). Only under Physioxia was the accumulation of collagen/DNA greater at the 7-week time point (Figure 3C).

**FIGURE 4:**
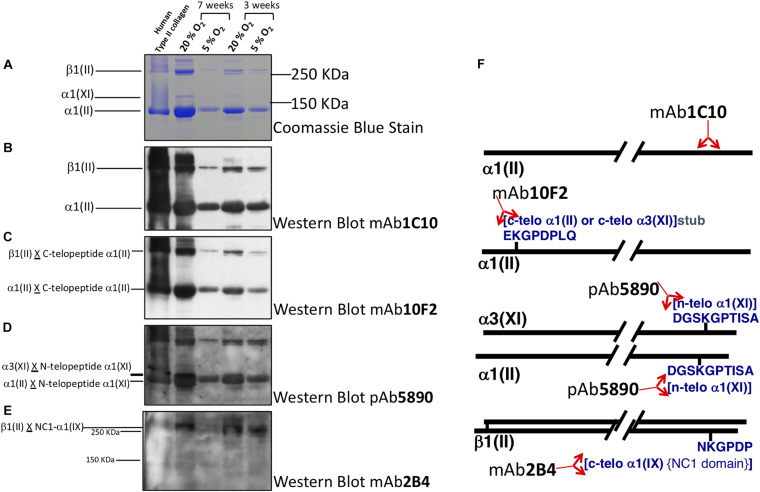
Collagen heteropolymer crosslink analysis. Collagen in human tissue-engineered cartilage was analyzed by SDS page and western blot. *Lane 1* human type II collagen (control); *Lane 2* 7-week Atm O_2_ (20%); *Lane 3* 7-week Physioxia (5%); *Lane 4* 3-week Atm O_2_; *Lane 5* 3-week Physioxia. Panels **(A)** Coomassie blue stain; **(B)** Western blot type using II collagen antibody to α1(II) chain in helical region; **(C)** Western blot using antibody to *C*-telopeptide of α1(II) chains of type II collagen; **(D)** Western blot using antibody to *N*-telopeptide of α1(XI) chains of type IX collagen; **(E)** Western blot of antibody that recognizes C-telopeptide of the non-collagenous domain of α1(IX) chains of type IX collagen of type IX collagen. **(F)** Locations of the epitopes in the α1(II) chain or in telopeptide stubs crosslinked to the collagen chains in type II-IX-XI heteropolymer.

Collagen trivalent HP and LP cross-links were formed in culture at both oxygen tensions and increased with time in culture, with no apparent effect of oxygen tension (Figure 3D). To determine if cartilage-specific collagen type II-IX-XI heteropolymer formed in culture we used specific antibodies to qualitatively fingerprint cross-linked collagen chains as we previously established (Murdoch et al., 2016). Figure 4A shows an SDS-PAGE gel of pepsin extracted collagen from human tissue engineered cartilage grown under Atm O_2_ and Physioxia. Purified human type II collagen (lane 1) and tissue engineered human cartilage is shown in the lanes 2–5. Only a qualitative evaluation of collagen in these samples was possible. The major Coomassie blue stained pepsin-resistant chain observed was the α1(II) collagen chain which migrates similarly to the chain seen for human type II collagen purified from adult articular cartilage. Two other pepsin-resistant chains of varying intensities were observed (lanes 2–5), migrating above the α1(II) chains. The chains migrate similarly to the α1(XI) and α2(XI) chains in type XI collagens. Faint bands in the region of α2(I) and β2(I) chain characteristic of type I collagen are seen in all lanes including the human type II control. Immunohistochemical analysis does reveal type I collagen in the matrix of the week 3 (Atm O_2_ and Physioxia) and week 7 (Atm O_2_) tissue engineered cartilage (Figure 6). Indeed, we have determined by mass spectrometry that type I collagen is a minor component of neo-cartilage from human bone marrow derived mesenchymal cells (Murdoch et al., 2016). A similar band was also observed in normal and chondrodysplastic human cartilage and was presumed to be the α2(I) chain (Fernandes et al., 1998). However, by *N*-terminal amino-acid sequencing the band was identified as a pepsin over-cleavage product of the α1(II) chain (Wu and Eyre, 1995; Fernandes et al., 1998).

Western Blot using the type II collagen antibody 1C10 (Figure 4B) confirmed the α1(II) and the β1(II) chains of type II collagen (lanes 2–5) indicating the chondrocytes elaborated an extensive extracellular matrix containing type II collagen. The antibody also recognized the band below β1(II) chains (distinct in lanes 4, 5 but obscured in lanes 1, 2 due to intense immunoreactivity of the β1(II) band) and the band below the α1(II) chains (lanes 4, 5) indicating these are over-cleavage products of the α1(II) chain. The antibody 10F2 reacted with the α1(II) chain, β1(II) chain and their over-cleavage products as expected for a cross-linked type II collagen polymer (Figure 4C), (*C*-telopeptide of type II collagen cross-linked to α1(II) collagen chains) indicating a cross-linked collagen network assembled in the all the neo-cartilages. Following an extended exposure, the antibody also reacted with α1(XI) collagen chains implying this chain was cross-linked to the *C*-telopeptide of type II collagen and that type XI collagen was copolymerized and cross-linked to *C*-telopeptides of type II collagen (data not shown). A similar blot when probed with the antibody 5890 clearly reacted with the α1(II) chain and the α3(XI) chain in the neo-cartilages (Figure 4D). (The α1(II) and α3(XI) chains are the identical product of the type II collagen gene but post translational modifications causes the chains to migrate differently on SDS-PAGE). This indicated that the *N*-telopeptide of the α1(XI) collagen chain was cross-linked to the α3(XI) and the α1(II) chain and thus a heteropolymer of type XI-type II collagen molecules had formed. The antibody 2B4 strongly reacted with the β1(II) chain of type II collagen in the neo-cartilages (Figure 4E) indicating that this chain was cross-linked to the α1(IX) chain of type IX collagen and a heteropolymer of type IX-type II had formed. This fingerprint pattern on western blots showed that a cross-linked heteropolymer of type II-IX-XI had assembled in the neo-cartilages. Figure 4F shows molecular interpretations of collagen heteropolymer assembly from Western blot analysis. Locations of the epitopes in the α1(II) chain or in telopeptide stubs cross-linked to the chains are also shown (McAlinden et al., 2014).

### Mechanical Assays

**FIGURE 5:**
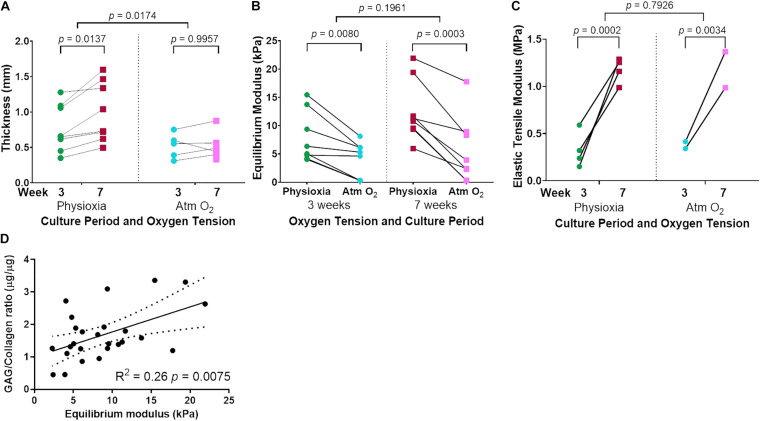
Mechanical testing of tissue-engineered human cartilage sheets. Tissue-engineered human cartilage sheet thickness over time (3- and 7-weeks) in both Physioxia and Atm O_2_ **(A)**. Compressive stiffness (Equilibrium Modulus) of tissue-engineered human cartilage sheets when grown in Physioxia vs Atm O_2_ both at 3- and 7-weeks **(B)**. Elastic tensile Modulus of tissue engineered human cartilage sheets in Physioxia and Atm O_2_ at 3- and 7-weeks **(C)**. Correlation of GAG/Collagen ratio with Equilibrium modulus **(D)**. **(A–C)** The average of data for each experiment is shown by a symbol with lines connecting a donor/experiment, **(A,B)** six donors, eight experiments, **(C)** 4 donors, 4 experiments. In **(D)** the symbols represent average sheet data regardless of the time or oxygen tension.

Tissue-engineered human cartilage sheet thickness increased with time in culture at both physiological and Atm O_2_ (Figure 5A). Sheets produced in Physioxia were significantly thicker than those produced in Atm O_2_ (Figure 5A). Compressive stiffness of the cartilage sheets was greater in sheets grown in Physioxia at both 3-weeks and 7-weeks (Figure 5B), time in culture was only a significant factor for sheets grown in Physioxia. Tensile stiffness of sheets increased with time in culture at both physiological and Atm O_2_ (Figure 5C), for this measure there was no appreciable effect of oxygen tension. The highest correlation for compressive mechanical stiffness with biochemical measures was achieved with the ratio of GAG/collagen (Figure 5D). None of the biochemical data showed significant correlation with elastic tensile modulus (Data not shown).

**FIGURE 6:**
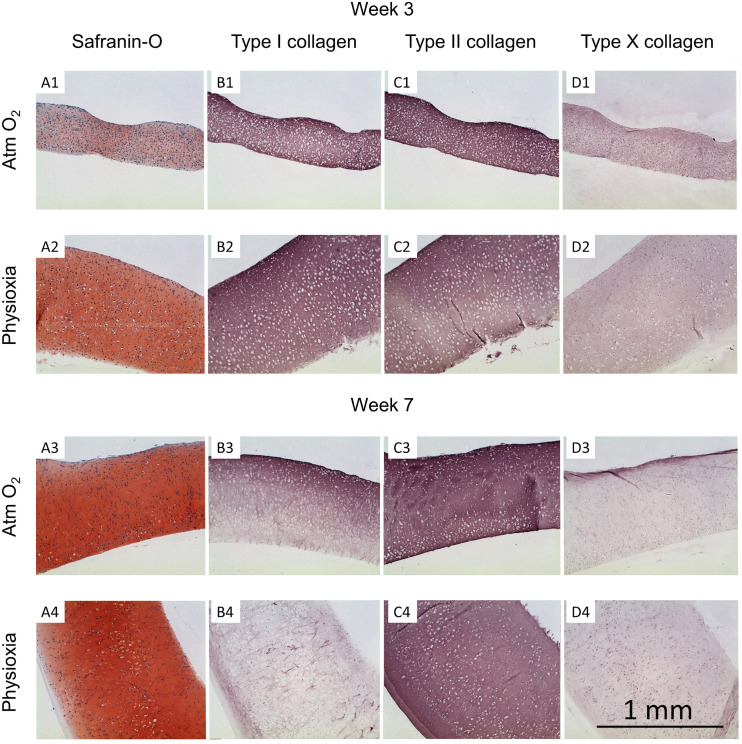
Histological analysis of human tissue-engineered cartilage sheets. Tissue-engineered human cartilage sheets from a single donor are shown (see Supplementary Figures 1,2 for other donors and Supplementary Figure 3 for native immunohistochemistry controls), they were stained for GAG (safranin-O, column **A**), type I collagen (column **B**), type II collagen (column **C**), and type X collagen (column **D**). Rows 1 and 2 show data from the 3-week time point and rows 3 and 4 show data from week-7. Atm O_2_ sheets are shown in rows 1 and 3, Physioxic sheets are shown in rows 2 and 4. The scale bar indicates a 1 mm distance.

### Histological Analyses

Tissue-engineered human cartilage sheets were stained for GAG content (safranin-O) and types I, II, and X collagen. At the 3-week time point, sheets were thicker when grown in physioxic conditions vs Atm O_2_ (0.77 ± 0.33 vs 0.52 ± 0.18 mm; mean ± S.D.; Figures 6 and 5A). Thickness increased over time in physioxic conditions but not in Atm O_2_ (7-week thickness 1.0 ± 0.42 vs 0.52 ± 0.19 mm Phsioxia vs Atm O_2_; mean ± S.D.). GAG staining was more intense in sheets grown under physioxic conditions (Figure 6A2) vs Atm O_2_ (Figure 6A1). Type I collagen and type II collagen staining was similar under both oxygen tensions at 3-weeks (Figures 6B1,B2,C1,C2). Type X collagen staining was slightly increased under Atm O_2_ (Figure 6D1) vs Physioxia (Figure 6D2) conditions at week 3. At the 7-week time point, safranin-O staining in sheets grown in Atm O_2_ (Figure 6A3) had increased to a similar level as sheets grown in physioxic conditions (Figure 6A4). Type I collagen staining under Atm O_2_ at week 7 (Figure 6B3) has decreased intensity vs the 3-week time point (Figure 6B1). Similarly, the sheets grown under physioxic conditions at week 7 have reduced or minimal staining for type I collagen (Figure 6B4) vs (Figure 6B2). Type II collagen was relatively intense under both oxygen tensions (Figures 6C3,C4). Type X collagen staining was more intense on the upper surface of the sheet grown under Atm O_2_ at week 7 (Figure 6D3). In sheets grown under physioxic conditions, type X collagen staining was predominantly intracellular vs. extracellular (Figure 6D4).

## Discussion

Critically, scaffold-free human tissue-engineered cartilage sheets were successfully formed in physioxic conditions for all donors. There was a large degree of variation between donors in terms of GAG deposition, collagen deposition and crosslink density. Even with this wide variation in donor response, a consistent effect of increased GAG deposition through growth under physioxic conditions was remarkable. Similarly, a consistent increase in total collagen deposition was also found through culture under physiological oxygen conditions. Unfortunately, the biochemical assay for collagen does not discriminate between the different types of collagen. This shortcoming is apparent when looking at the histological data, where temporal and regional variation in collagen type and intensity are evident. Initial expression and replacement of type I collagen has been documented developmentally *in vivo* (Morrison et al., 1996; Sasano et al., 1996) and, as we have also shown in human bone marrow derived stem cell neo-cartilage, engineered *in vitro* (Murdoch et al., 2016). This could indicate that expression and replacement is a normal progression in tissue-engineered cartilage development and that replacement of type I with type II collagen is aided by Physioxia. Growth on synoviocyte matrix in the absence of any synthetic scaffold allows for significant population doublings while retaining chondrogenic capacity, enough that large (16 cm^2^) pieces of tissue engineered human cartilage can be grown (Figure 3A). There was no apparent effect of oxygen tension on trivalent collagen cross-linking, but significant increases in total HP and LP cross-link formation were observed with longer culture duration. This indicates that under both physioxic and normoxic conditions a fibrillar network of collagen with mature cross-links had formed in neo-cartilage. Western blot analyses using established antibodies to specific collagen peptides involved in covalent cross-link formation (Fernandes et al., 2003; McAlinden et al., 2014) indicated that a cross-linked hetropolymer of type II-IX-XI collagen had formed in the tissue engineered cartilage. This cross-linked collagen heteropolymer is typical of cartilage and is essential in the proper assembly of the cartilage collagen fibril (Eyre et al., 2002). Our findings that a similar nascent heteropolymeric template is formed in human neo-cartilage with increased cross-linking with time in culture point to a progressive formation of type II collagen based fibril network typical of cartilage.

There are multiple mechanisms by which Physioxia is thought to increase GAG, collagen deposition and biomechanics. An excellent review by Pattappa et al. (2019) highlights the many mechanisms that have been identified in MSC chondrogenesis. Briefly, they identified inhibition of IL1B effects, upregulation of TGFβ receptors, HIF stabilization and expression and stimulation of SOX9 (Pattappa et al., 2019). It is likely that some, if not most, of these factors would be mechanisms by which tissue engineered cartilage derived from articular chondrocytes would benefit. Others have focused on the hypoxia inducible factors HIF1α, HIF2α and HIF3α, and HIF3α was found to be particularly important for cartilage health from both MSCs and chondrocytes (Markway et al., 2015).

Subjective assessment of the sheets (physical handling) indicate that longer culture durations gave stronger sheets in all cases, although this was not fully supported by the equilibrium moduli which only showed a benefit in sheets grown under Physioxia. This is potentially due to untestable sheets formed under Atm O_2_ culture (5 of 16 sheets). However, the results did show an increase in both total collagen content and collagen cross-link content with time. These increases correlated with the increased tensile properties of the tissues. Makris et al. (2013b) also found that hypoxia (4% O_2_) increased collagen crosslinks in tissue engineered bovine cartilage constructs but that they also found weak correlations to compressive mechanical properties. The combined increase in compressive and tensile stiffness, along with the greater accumulation of extracellular matrix components, through culture under Physioxia leads us to recommend this culture method for chondrogenic experiments.

While autologs chondrocytes have many benefits, without a mechanism to improve poor responders they may not be the best cell choice for tissue engineered grafts. Indeed, the relatively immunopriveledged site of the joint does seem to accept grafts without the need for any HLA matching. This creates an opportunity for a well characterized donor pool of chondrocytes to be created to make tissue engineered cartilage grafts. There remain significant challenges in producing autologs, tissue-engineered cartilage with sufficient biomechanical properties to be implanted as a functional replacement. Current clinical trials cover a wide range of approaches, many using allogenic cells (for review see Kwon et al., 2019). The advantages of a well characterized allogenic cell bank are clear given the range of GAG and collagen contents due to donor variability. In chondrocyte progenitor experiments, this has been further focused in on showing clonal variability (Anderson et al., 2016). Interestingly, while there was a wide range of extracellular matrix component concentrations detected, the distribution in mechanical properties was actually relatively narrow. Evans and Quinn (2005) found that cartilage compressive stiffness correlated with GAG density in native tissues. Roeder et al. (2002) found that tensile modulus increased with increasing concentration of type I collagen engineered constructs in a linear manner. Our data showed the greatest correlation of biochemical measures with compressive moduli when the GAG/Collagen ratio was used but, probably due to the four conditions and six donors, this correlation was relatively weak. When looking at biochemical content correlations with tensile properties, nothing gave a significant correlation; this analysis was hampered by the number of conditions analyzed and lack of sheet formation for two of the four donors at Atm O_2_. Similarly, Williamson et al. (2003) found no significant correlation between bovine fetal cartilage and tensile tests. Overall, the data indicate that physiological oxygen tension is beneficial to chondrogenesis of human tissue-engineered cartilage sheets formed through scaffold-free culture of human articular chondrocytes.

Another issue to be addressed is the timing of implantation of tissue-engineered sheets. The methods used in this study pre-suppose the formation of a rigid cartilage matrix prior to implantation, which is a different approach to that of many others (Kwon et al., 2019). This raises the issue of the timing of implantation. If the implant is made too early, the construct may be compromised by the loads experienced *in vivo*. Alternatively, if the material is too stiff, there may be problems related to the integration into the underlying bone or along the lateral edges. One approach is to engineer the constructs such that the lateral edges are in a non-load or minimally-loaded area. Alternatively, it may be necessary to engineer the edges differently so that they might withstand tensional forces of suture material.

## Conclusion

Tissue-engineered human cartilage sheets, formed through scaffold free self-assembly of articular chondrocytes, in Physioxia have significantly more extracellular matrix with correlative increases in compressive stiffness than sheets grown under Atm O_2_. The combination of Physioxia with longer culture duration resulted in the formation of the type II-IX-XI collagen heteropolymer with greater collagen crosslinks vs sheets grown in Atm O_2_ conditions.

## Data Availability Statement

The original contributions presented in the study are included in the article/Supplementary Material, further inquiries can be directed to the corresponding author.

## Author Contributions

TK, JD, GW, and RF: concept and manuscript writing. TK, JD, GW, and RF: design, data analysis and interpretation, and manuscript editing. TK, JD, and RF: financial support. TK, GW, JR, and RF: collection of the data. TK, JD, GW, and RF: final approval of the manuscript. All authors contributed to the article and approved the submitted version.

## Conflict of Interest

The authors declare that the research was conducted in the absence of any commercial or financial relationships that could be construed as a potential conflict of interest.

## Supplementary Material

Supplementary Figure 1 Week 3 histology images of donors (B,C,D) sheets produced under Physioxia and Atm O_2_. 1st passage sheets are p1 and second passage sheets are p2.

Supplementary Figure 2 Week 7 histology images of donors (A,B,C,E) sheets produced under Physioxia and Atm O_2_. 1st passage sheets are p1.

Supplementary Figure 3 Immunohistochemistry controls, type I collagen showing staining in the perichondrium of the rabbit (Rb) ear and in the bone of the Rb Knee. Type II collagen showing staining in the cartilage of the Rb ear, human (Hu) knee and Rb knee articular and growth plate cartilage. Type X collagen showing staining in the Rb Ear, and Rb knee hypertrophic and calcified growth plate cartilage.

Supplementary Data Sheet 1 Template for printing overhead projector sheets to create clamps for the cartilage tissue tension tests. Dotted line indicates where the testing machine clamps should go, window in center is cut out with craft knife or scalpel, tissue adhered with dog bone over window, kept hydrated until placed in testing device then edges of the window cut before applying load.
