# Supplementary figures and images for: Physioxia Stimulates Extracellular Matrix Deposition and Increases Mechanical Properties of Human Chondrocyte-Derived Tissue-Engineered Cartilage
Source: Front Bioeng Biotechnol. 2020 Nov 13;8:590743. doi: 10.3389/fbioe.2020.590743 (PMC7691651; doi:10.3389/fbioe.2020.590743)

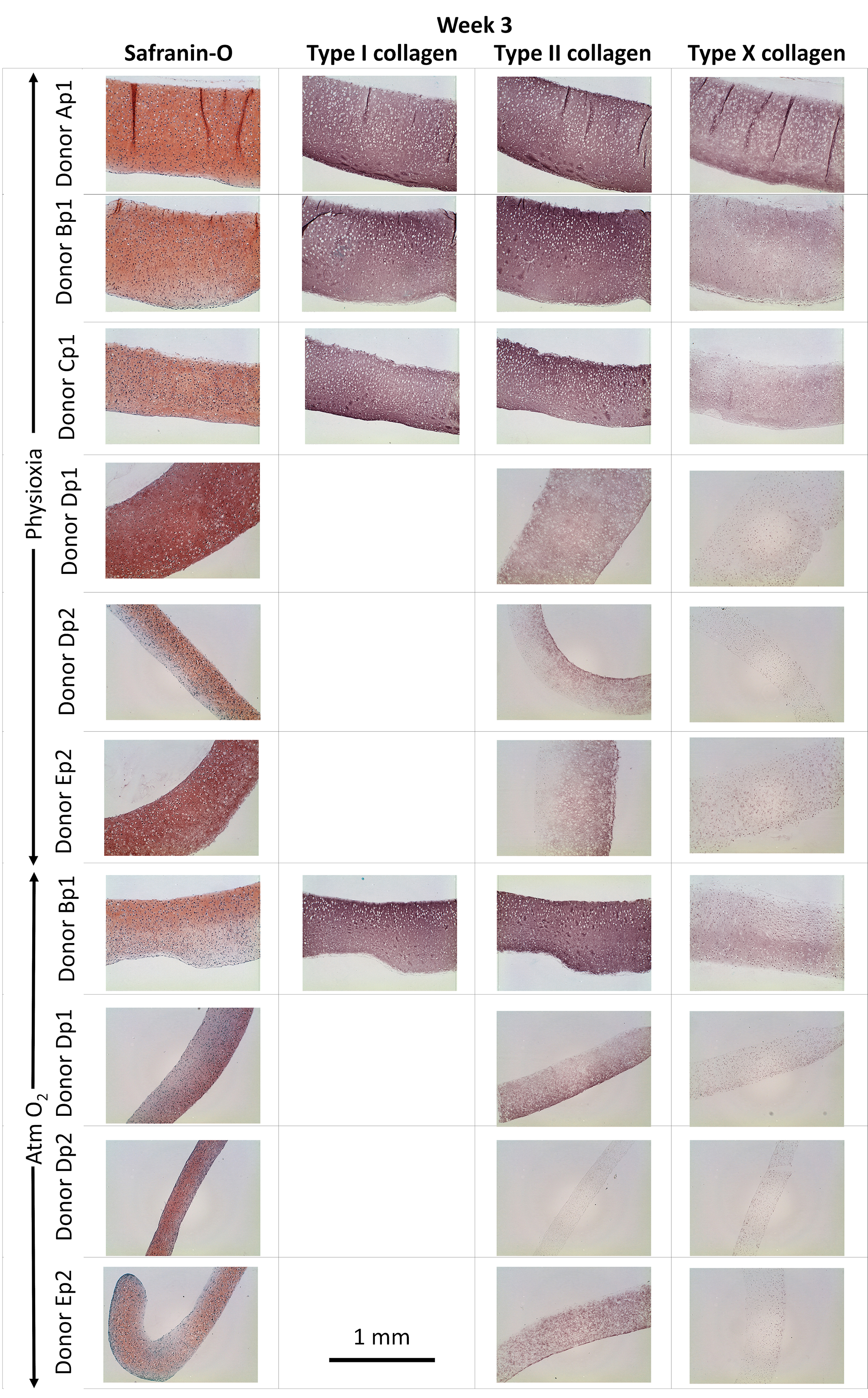

Supplement: Supplementary Figure 1 — Week 3 histology images of donors (B,C,D) sheets produced under Physioxia and Atm O2. 1st passage sheets are p1 and second passage sheets are p2. [file Image_1.TIF]

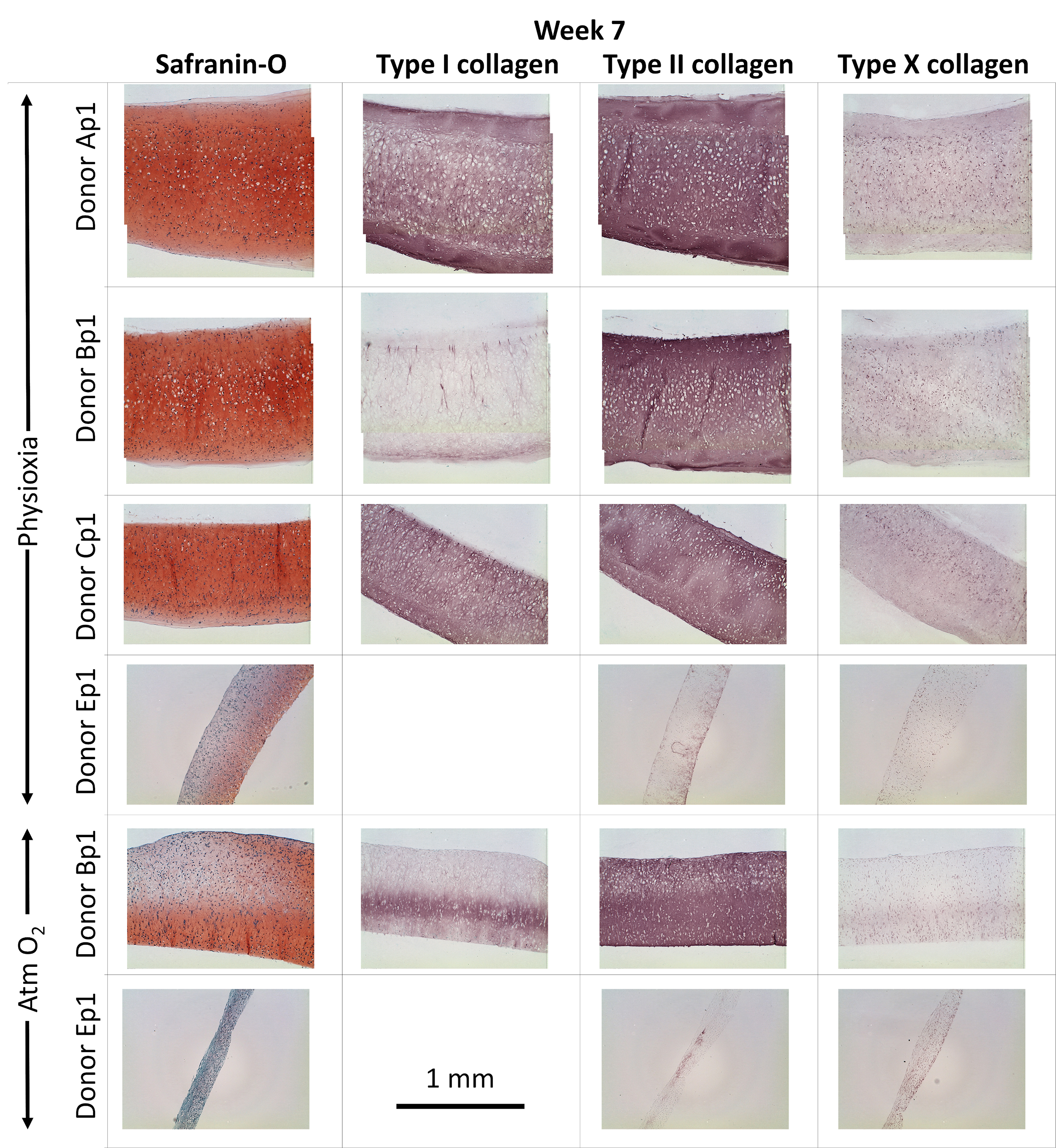

Supplement: Supplementary Figure 2 — Week 7 histology images of donors (A,B,C,E) sheets produced under Physioxia and Atm O2. 1st passage sheets are p1. [file Image_2.TIF]

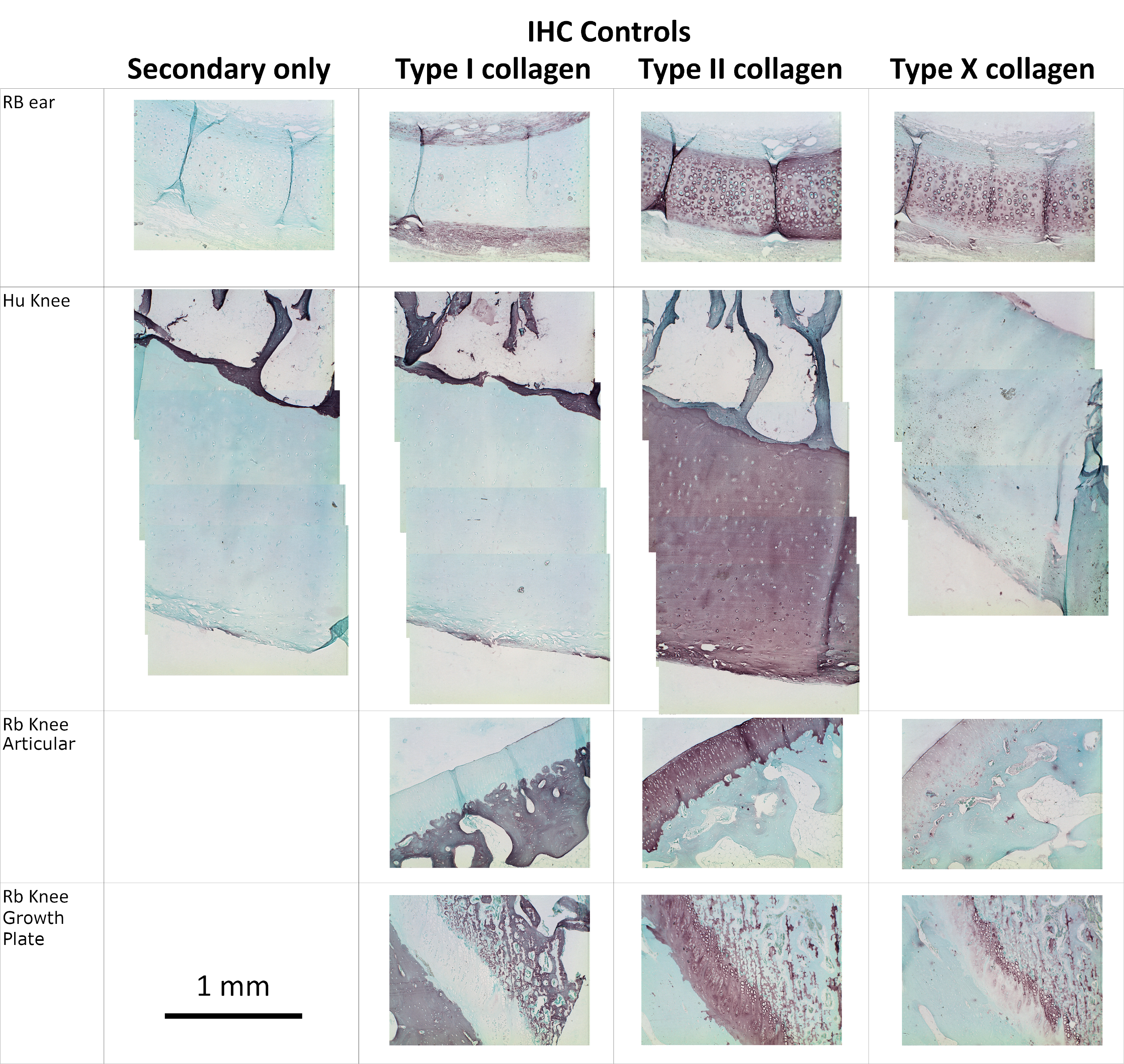

Supplement: Supplementary Figure 3 — Immunohistochemistry controls, type I collagen showing staining in the perichondrium of the rabbit (Rb) ear and in the bone of the Rb Knee. Type II collagen showing staining in the cartilage of the Rb ear, human (Hu) knee and Rb knee articular and growth plate cartilage. Type X collagen showing staining in the Rb Ear, and Rb knee hypertrophic and calcified growth plate cartilage. [file Image_3.tif]
